# Primary Burkitt lymphoma of the thyroid gland: case report of an exceptional type of thyroid neoplasm and review of the literature

**DOI:** 10.1186/s12907-016-0028-6

**Published:** 2016-05-11

**Authors:** Mohamed Allaoui, Ilias Benchafai, El Mehdi Mahtat, Safae Regragui, Adil Boudhas, Mustapha Azzakhmam, Mohammed Boukhechba, Abderrahmane Al Bouzidi, Mohamed Oukabli

**Affiliations:** Department of Pathology, Military General Hospital Mohammed V, Mohammed V Souissi University - Faculty of Medicine and Pharmacy of Rabat, Hay Riad, Rabat, 10000 Morocco; Department of Clinical Haematology, Military General Hospital Mohammed V, Mohammed V Souissi University - Faculty of Medicine and Pharmacy of Rabat, Hay Riad, Rabat, 10000 Morocco; Department of Otorhinolaryngology, Military General Hospital Mohammed V, Mohammed V Souissi University - Faculty of Medicine and Pharmacy of Rabat, Hay Riad, Rabat, 10000 Morocco

**Keywords:** Burkitt lymphoma, Thyroid gland, Chemotherapy

## Abstract

**Background:**

Primary thyroid lymphoma is an uncommon pathological entity that accounts for only 1 to 5 % of all thyroid malignancies. Primary Burkitt lymphoma of the thyroid gland is very rare. This article presents the first Moroccan case of a primary BL of the thyroid to be reported in the literature to date.

**Case presentation:**

We describe here a case of a 70-year-old male who developed a rapidly enlarging thyroid gland with progressive symptoms of compression. Core biopsy confirmed the diagnosis of Burkitt lymphoma. The patient died of septic shock, 2 weeks after the first cycle of appropriate therapeutic chemotherapy.

**Conclusions:**

This presentation emphasizes the importance of considering lymphoma when dealing with a thyroid mass, as its management is different from that of other thyroid pathologies, and affords an opportunity to review a very rare type of primary thyroid lymphoma.

## Background

Primary Burkitt lymphoma (BL) of the thyroid gland is a very uncommon pathological entity with a few isolated case reports in adult patients [1, 2].

This highly aggressive malignancy arises from B-lymphoid cells. It presents usually as a rapidly expanding thyroid mass causing compressive symptoms.

To the best of our knowledge, this article reports the first Moroccan case of a primary BL of the thyroid to be reported in the literature to date.

BL should be promptly recognized because its management is quite different from the treatment of other neoplasms of the thyroid gland. Moreover, this disease is quite curable if diagnosed early and treated appropriately.

## Case presentation

A 70-year-old male presented a rapidly expanding mass of the neck associated with history of airway compression symptoms; progressive dyspnea and dysphonia lasting for 4 weeks, in a context of apyrexia and impairment of general condition. The patient was admitted to the hospital because of increasing dyspnea and urgently received a tracheostomy.

**Fig. 1:**
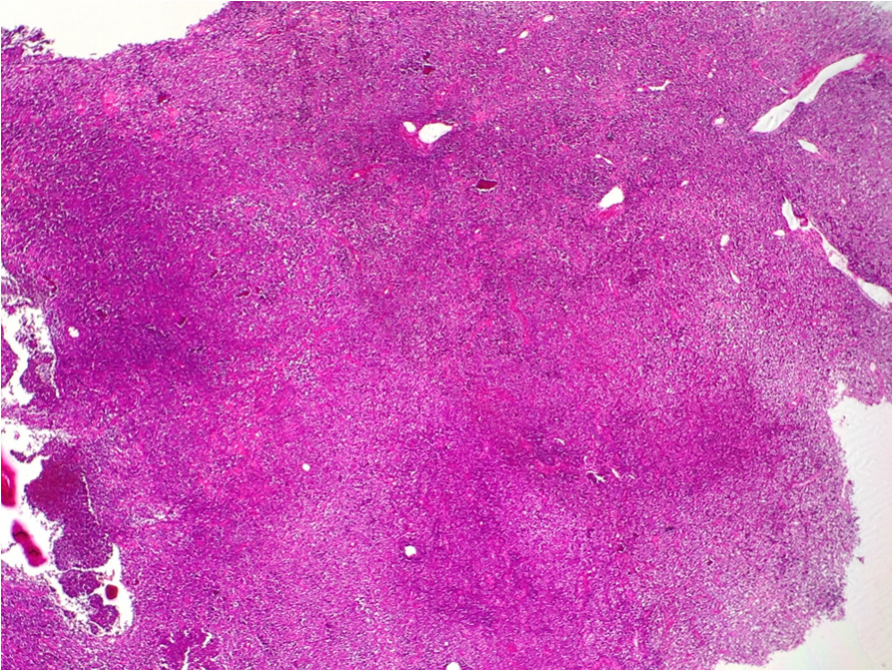
Low magnification showing a diffuse infiltration of atypical lymphoid cells in the thyroid gland (haematoxylin & eosin stain, ×50)

**Fig. 2:**
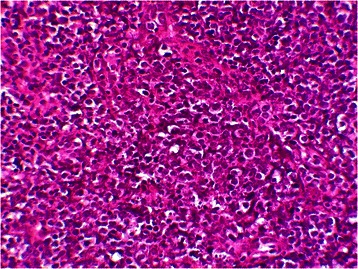
Higher magnification showing a monotonous population of intermediate-sized lymphoid cells with scant dark blue cytoplasm, small cytoplasmic vacuoles and tingible-body macrophages (haematoxylin & eosin stain, ×400)

A biopsy of the cervical mass was carried out and the histological examination showed diffuse infiltration of the thyroid gland by a monotonous population of atypical intermediate-sized lymphoid cells (Fig. 1). These last possess scanty amphophilic to basophilic cytoplasm with centrally located nuclei of irregular shape, displaying dispersed basophilic chromatin, and frequent apoptotic figures (Fig. 2). Scattered tingible body type macrophages were also present. Little residual thyroid follicles and some areas of necrosis was observed.

**Fig. 3:**
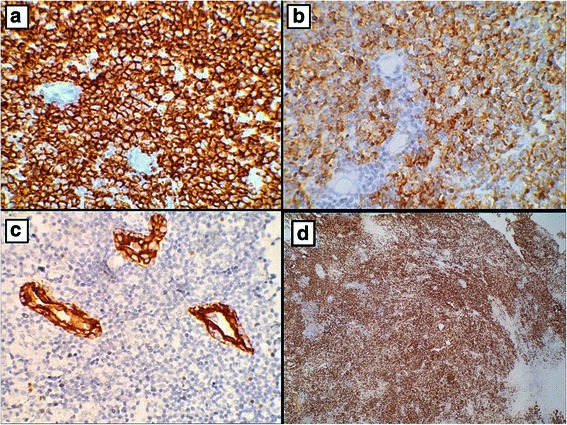
Immunohistochemical staining revealed the expression of CD20 (**a**) and CD10 (**b**) by the neoplastic cells. Keratin highlights the lymphoepithelial lesions (**c**). Ki-67 immunostaining showed a high proliferation index (**d**)

Immunohistochemical staining was then performed and the tumour cells were positive for CD20, CD10 and BCL6. Ki-67 showed proliferation index approaching 100 %. CD3 and CD5 stained the background T cells (Fig. 3). Immunoreactivity for Epstein-Barr virus (EBV) was negative. The diagnosis of BL was confirmed on fluorescence in situ hybridisation that showed tumour cell positivity for the t (8; 14) translocation. Bone marrow examination was normal.

The patient was transferred to the Clinical Haematology department. On physical examination, he was apyretic and hemodynamically stable with a cervical armouring by a huge mass of hard consistency. Neurological examination shows no sensorimotor deficits.

Other systemic examinations were normal, without any palpable lymphadenopathy or organomegaly.

**Fig. 4:**
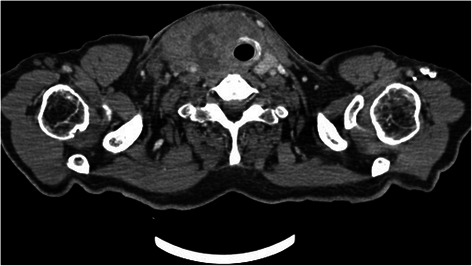
CT showing a large heterogeneous mass of the thyroid gland

The computerized tomography (CT) scan showed a heterogeneous process of the thyroid gland measuring 10.5 × 8.2 × 6.5 cm in size, extending up towards the laryngeal region, infiltrating the right vocal cord and reducing the laryngeal lumen (Fig. 4).

The thoraco-abdominal CT scan showed no other localization.

The examination of the cephalorachidian liquid showed no central nervous system involvement. The patient was diagnosed with thyroidal Burkitt lymphoma, stage I of Murphy [3].

After cardiac, renal and liver functions assessment, the patient received chemotherapy according to the LMBA02 protocol [4], group A (Age > 60 years, no CNS nor bone marrow involvement), with a first course of COP (Cyclophosphamide, Vincristine and Prednisone) and intrathecal Methotrexate, followed a week after by a course of R-COPADEM (Rituximab at day 0 and day 6, Cyclophosphamide day 2,3 and 4, Prednisone from day 1 to 5, Doxorubicineat day 2, high dose Methotrexate at day 1 with folic acid rescue from day 2 to day 6 and intrathecal chemotherapy at day 2 and day 6).

At day 15 of chemotherapy, the patient developed febrile neutropenia, refractory to broad-spectrum antibiotics and antifungals. The evolution was marked by the installation of a septic shock with acute respiratory distress syndrome that led to his transfer to intensive care unit where he was intubated and ventilated. The patient died 2 days after.

## Discussion

Primary thyroid lymphoma (PTL) is defined as a lymphoma involving only the thyroid gland or the thyroid gland and adjacent (regional) neck lymph nodes, without contiguous spread or distant metastases from other areas of involvement at diagnosis [5].

PTL is an uncommon pathological entity that accounts for only 1 to 5 % of all thyroid malignancies, comprises approximately 2 % of all malignant extra nodal lymphomas and which predominantly originate from B lymphocytes [5, 6].

The common histological subtypes are diffuse large B-cell lymphoma and mucosa-associated lymphoid tissue (MALT) lymphoma [6–9]. Primary Burkitt lymphoma of the thyroid is very rare with a few isolated case reports (Table 1) [1–3, 10, 11].

**Table 1:** Clinical and pathological characteristics of patients with primary Burkitt lymphoma of the thyroid gland in the literature

Author	Age (year)	Sex	Clinical presentation	Size of tumor (cm)	Histology	Translocation type	Treatment	Follow-up time (month)	Evolution
Our case	70	M	Rapidly expanding mass of the neck associated with airway compression symptoms	10.5	Burkitt lymphoma	t (8; 14)	chemotherapy according to the LMBA02 protocol; with a first course of COP and intrathecal Methotrexate, followed by a course of R-COPADEM.	Patient died	The patient died of septic shock, 2 weeks after the first cycle of chemotherapy
Camera et al. [2010] [1]	56	M	Incidental discovery of a large left thyroid lobe nodule on CT	4.9	Burkitt-like large B–cell lymphoma		Left lobe thyroidectomy. After diagnosis, The patient was treated with 8 cycles of intensive chemotherapy (cyclophosphamide, vincristine, doxorubicine, and dexamethasone)	1	Reduction of all lesions with improvement of symptoms.
Kalinyak et al. [2006] [2]	53	M	Tracheal compressive symptoms from a rapidly expanding thyroid mass	6	Burkitt lymphoma		Rituxan and CHOP therapy, changed to hyper-CVAD-R chemotherapy. The patient also received a single dose of intrathecal methotrexate	27	Patient free of disease after end of treatment
Kandil et al. [2012] [10]	60	F	Rapidly expanding thyroid mass with airway compression and difficulty in swallowing	8.7	Burkitt-like lymphoma (B-cell lymphoma, unclassifiable)		Rituximab, Cyclophosphamide, Mensa, Vincristine and Doxorubicin		Successfully treated with 1 cycle of appropriate therapeutic chemotherapy
Cooper et al. [2014] [14]	14	M	Large predominantly left-sided firm thyroid swelling, with a 3-month history of malaise, lethargy, and weight loss	6.7	Burkitt lymphoma	t (8; 14)	COP and prednisolone followed by 2 courses of COPADM, prednisolone and two courses of CYM chemotherapy. This was accompanied by intrathecal chemotherapy	36	Disease free 3 years after end of treatment
Yildiz et al. [2012] [22]	31	M	Rapidly enlarging mass on the fore neck	4	Burkitt lymphoma		R-Hyper-CVAD therapy	6	PET-CT scans performed after chemotherapy and at the 6-month follow-up were normal
Mweempwa et al. [2013] [24]	58	F	Background of benign goiter presented with a rapidly enlarging thyroid mass, causing dysphagia and dyspnea	8	Burkitt lymphoma	t (8; 14)	Modified Magrath protocol for Burkitt’s lymphoma, low risk disease, which involved having 3 cycles of R-CODOX-M	4	Complete resolution of the tumour mass, 4 weeks after end of treatment
Liying et al. [2014] [30]	8	M	Mass in the right anterior neck with difficulty in swallowing	4	Burkitt lymphoma	t (8; 14)	Right lobe thyroidectomy. After diagnosis, the patient underwent alternate R-B-NHL-BFM-90-A and R-B-NHL-BFM-90-B treatment, for 4 cycles each	48	After 4 years of follow-up, the patient appears well and remains free of disease

Burkitt lymphoma is a highly aggressive disease that is endemic in Africa and sporadic in other parts of the world. The endemic variant is associated with Epstein-Barr virus.

BL was one of the first tumours shown to have a chromosomal translocation that activates an oncogene (*c-MYC*) [12, 13].

Normally, the thyroid gland does not contain native lymphoid tissue, therefore, the intrathyroid lymphoid tissue that causes thyroid lymphoma comes from the migration of lymphoid tissue into the thyroid during an inflammatory or immunologic process. The most common condition resulting in lymphoid migration is autoimmune thyroiditis (i.e., Hashimoto’s thyroiditis) [14–16]. Large adult population-based as well as retrospective clinicopathological case series suggest that primary thyroid Non-Hodgkin lymphoma NHL typically occur in middle to older-aged persons and have a predilection for females (it have also shown that patients with chronic lymphocytic thyroiditis have a greater risk of subsequently developing thyroid lymphoma when compared to age and gender matched normal individuals) [5, 6, 16–21].

Clinically, lymphomas originating in the thyroid can frequently mimic anaplastic thyroid carcinoma in that both have similar clinical characteristics of rapid growth, which might be associated with compression symptoms dyspnea, dysphagia, pain and hoarseness of voice [2, 22–25].

Ultrasonography is generally the initial diagnostic modality used in the workup of thyroid enlargement and nodules. Although nonspecific, there are certain characteristics that suggest PTL, based essentially upon ultrasound findings of internal echoes, borders, and posterior echoes. For example, Enhanced posterior echoes help in distinguishing lymphoma from other types of thyroid lesions [26, 27].

Once PTL is suspected based upon clinical presentation and ultrasound findings, the next step in diagnosis is biopsy. Traditionally, open surgical biopsy was felt to be necessary to differentiate thyroid lymphoma from thyroiditis and anaplastic carcinoma. However, with recent advances in immunophenotypic analysis, the accuracy of fine-needle aspiration (FNA) has improved. These advances in diagnosis of PTL mirror those seen with systemic lymphomas with a reported accuracy rate of FNA of 80–100 %. Nevertheless, there are still challenges in FNA diagnosis of thyroid lymphoma, particularly due to the histological similarities with thyroiditis and the high coincidence of these pathologies within the same gland, which results in increased false-negative rates from sampling error [26].

Histologically, the tumour cells of BL are medium-sized cells (nuclei similar or smaller to those of histiocytes) and show a diffuse monotonous pattern of growth. The cells appear to be cohesive but sometimes exhibit squared-off borders of retracted cytoplasm. The nuclei are round with finely clumped and dispersed chromatin, with multiple basophilic medium-sized, paracentrally situated nucleoli. The cytoplasm is deeply basophilic and usually contains lipid vacuoles. The tumour has an extremely high proliferation fraction (many mitotic figures) as well as a high fraction of apoptosis. A “starry sky” pattern is usually present, which is imparted by numerous benign macrophages that have ingested apoptotic tumour cells [28].

In Burkitt lymphoma, the tumour cells express moderate to strong levels of membrane IgM with light chain restriction and B-cell-associated antigens (CD19, CD20 and CD22), CD10, BCL6, c-MYC and CD38. The neoplastic cells are usually negative or only weakly positive for BCL2 and are uniformly TdT and MUM-1/IRF-4 negative. Nearly 100 % of the cells are positive for Ki67. There are very few admixed T-cells [12, 28–30]. In the present case, the immunophenotype of the atypical lymphoid cells was consistent with these features.

Burkitt lymphoma is characterized at the molecular level by a reciprocal translocation involving the *c-MYC* proto-oncogene, which normally resides on chromosome 8q24. The most common translocation in Burkitt lymphoma is a t (8;14) (q24;q32), which results in the translocation of *c-MYC* to the B-cell heavy-chain gene locus on chromosome 14q32. This translocation occurs in approximately 80 % of Burkitt lymphoma cases regardless of the clinical setting. Other variant translocations involve the translocation of *c-MYC* to the kappa light chain locus on chromosome 2, t (2;8) (p12;q24), which occurs in approximately 15 % of cases, and translocation of *c-MYC* to the lambda light-chain locus on chromosome 22, t (8;22) (q24;q11), which occurs in approximately 5 % of cases [12, 28, 31].

In addition, it has been confirmed that the occurrence of BL is associated with viral infections, particularly EBV infection. However, the EBV detection rates in different subtypes of BL also vary [32]. EBV infection is detected in the vast majority of endemic BL and ~30 % of sporadic BL [12, 30, 32].

Treatment of Burkitt lymphoma in most centers is guided by the FAB LMB study (cooperative study between the Children’s Cancer Group, the Societé Francaise d’Oncologie Pediatrique, and the UK Children’s Cancer Study Group) [33, 34]. The former consists of initial cytoreduction with cyclophosphamide, prednisolone, and vincristine, followed by more intensive chemotherapy in varying combinations containing doxorubicin, alkylators, vincristine, etoposide and therapy directed to eradicate or to prevent CNS disease such as high dose methotrexate [3, 12].

Aggressive high-dose therapy is needed for adult Burkitt lymphoma. However, interpretation of response is difficult because most studies of this approach have been done with a single protocol in mainly young adults. The regimen generally used in the UK and USA is cyclophosphamide, vincristine, doxorubicin, and highdose methotrexate alternating with ifosfamide, etoposide, and high-dose cytarabine [12].

The use of a less toxic dose adjusted EPOCH (etoposide, prednisone, vincristine, cyclophosphamide, adriamycin) plus rituximab (DA-REPOCH) led to an event free survival of 96 % and an overall survival of 100 % with a median follow up to 86 months in a small studay that included 19 HIV negative patients [35]. The use of rituximab (anti-CD20) in primary therapy has been assessed and a randomized clinical trial including 257 patients demonstrated that the addition of rituximab to the LMB regimen improved the event free survival and the overall survival without adding more toxicities [4].

## Conclusions

In summary, the current study presents a case of sporadic primary BL of the thyroid occurring in a seventy-year-old male, which exhibited the typical morphological features and immunophenotype of BL and which, to the authors’ knowledge, is the first case of BL of the thyroid gland to be reported in Morocco. We also emphasize here the importance of considering lymphoma when dealing with a thyroid mass, as its management is different from that of other thyroid pathologies and delaying treatment has an impact on prognosis.

## Consent

Written informed consent was obtained from the patient’s family for publication of this case report and any accompanying images. A copy of the written consent is available for review by the Editor of this journal.

## Ethics approval and consent to participate

Not applicable.

## Abbreviations

BL: Burkitt lymphoma
CT: computerized tomography
EBV: epstein-barr virus
PTL: primary thyroid lymphoma
